# Cognitive flexibility, technostress, and mathematics teachers' acceptance of ChatGPT: a cross-sectional indirect-effect analysis

**DOI:** 10.3389/fpsyg.2026.1991429

**Published:** 2026-10-08

**Authors:** Baris Demir, Rumeysa Beyazhancer

**Affiliations:** 1 Department of Mathematics Education, Kocaeli University, Izmit, Türkiye; 2 Department of Mathematics Education, Bursa Uludag Universitesi, Nilüfer, Türkiye

**Keywords:** ChatGPT, cognitive flexibility, general technology-related technostress, generative artificial intelligence, mathematics teachers, technology acceptance

## Abstract

**Introduction:**

Generative artificial intelligence (GenAI) is rapidly entering mathematics classrooms, yet little is known about how teachers psychologically adapt to its use. Drawing on the Technology Acceptance Model (TAM) as an organizing framework and the transactional theory of stress and coping as a process account, this study examined whether general technology-related technostress statistically accounted for part of the association between cognitive flexibility and ChatGPT acceptance.

**Methods:**

This cross-sectional correlational study included 127 in-service middle-school mathematics teachers from 30 public schools in the Marmara Region of Türkiye. Validated self-report instruments assessed cognitive flexibility and general technology-related technostress. ChatGPT acceptance was calculated from perceived usefulness, perceived ease of use, attitude toward use, and pedagogical AI self-efficacy; actual use was excluded. A regression-based indirect-effect analysis (PROCESS Model 4; 5,000 bootstrap samples) controlled for gender.

**Results:**

Cognitive flexibility was negatively associated with general technology-related technostress (B = −0.243, *p* < 0.001), which was negatively associated with ChatGPT acceptance (B = −0.620, *p* < 0.001). Cognitive flexibility was positively associated with ChatGPT acceptance after technostress was included (B = 0.355, *p* < 0.001). The bootstrapped indirect effect was positive (B = 0.151, SE = 0.043, 95% CI [0.074, 0.241]).

**Discussion:**

Teachers reporting greater cognitive flexibility also reported lower general technology-related technostress and greater ChatGPT acceptance. Because all variables were measured concurrently by self-report, the indirect effect is statistical and does not establish temporal or causal mediation. The findings identify psychological adaptation as a relevant complement to technology-centered accounts of GenAI acceptance and motivate longitudinal research with practicing teachers.

## 1 Introduction

Generative artificial intelligence (GenAI) technologies are being introduced into educational settings before a mature evidence base has emerged to guide their use. Tools such as ChatGPT can generate explanations, examples, feedback, and lesson materials, but their instructional value depends partly on how teachers evaluate and manage their use (Kasneci et al., 2023; Lo, 2023; Rahman and Watanobe, 2023).

Specifically in mathematics teaching, ChatGPT and similar tools have been used to support lesson planning, generate sample problems, and produce conceptual explanations; however, their mere availability does not guarantee effective pedagogical adoption. In the context of mathematics teacher education, adoption has been associated with perceived usefulness, perceived ease of use, attitudes toward use, and pedagogical AI self-efficacy (Demir, 2026). In mathematics education, teachers must not only learn a new interface; they must also judge the accuracy of generated mathematical content, decide when GenAI assistance is pedagogically appropriate, and retain responsibility for instructional decisions. These demands make teachers' responses to GenAI simultaneously technological, cognitive, and emotional. The present study, therefore, focuses on two psychological correlates of ChatGPT acceptance—cognitive flexibility and technostress—among practicing middle-school mathematics teachers.

Teachers are central to whether GenAI becomes a meaningful instructional resource rather than an intermittently used technical novelty, because their acceptance is closely related to the adoption and use of digital technologies in teaching (Scherer et al., 2019; Tan, 2026; Wang et al., 2025). Yet acceptance cannot be understood only as a response to a tool's technical features. GenAI requires teachers to interpret unfamiliar outputs, revise established practices, manage uncertainty, and respond to new workload and professional-role demands (Kasneci et al., 2023; Wu et al., 2026). Qualitative evidence from secondary mathematics teachers likewise shows that perceived instructional opportunities coexist with concerns about mathematical inaccuracies, visual representations, and pedagogical fit (Busuttil and Calleja, 2025). Consequently, teachers' engagement with GenAI also involves cognitive adaptation and technology-related stress.

Against this background, the present study examines cognitive flexibility and general technology-related technostress as two interrelated psychological correlates of mathematics teachers' acceptance of AI. Cognitive flexibility may be relevant because teachers must consider alternative uses, reconsider unsuccessful interactions, and adapt instructional decisions when AI outputs are incomplete or unsuitable. Existing findings associate cognitive flexibility with lower technostress in general ICT use and with more favorable AI-related responses, including higher AI acceptance and lower AI anxiety (Bali et al., 2024; Kaya and Keskin, 2026). Technostress may be relevant because continued learning demands, uncertainty, workload, and reduced perceptions of control can accompany rapidly changing technologies; research among teachers also identifies competence, role conflict, organizational support, and technological characteristics as important correlates of AI-related technostress (Wu et al., 2026). However, evidence concerning technostress and AI acceptance is not uniform: Çakir and Güner (2026) found that technostress did not significantly predict teachers' AI acceptance. The present study, therefore, tests whether general technology-related technostress statistically accounts for part of the association between cognitive flexibility and global ChatGPT acceptance among practicing middle-school mathematics teachers. By integrating an adaptive cognitive resource with a technology-related strain response, the study complements technology-centered acceptance models and provides a subject-specific account of teachers' psychological adaptation to GenAI. The following section develops this rationale and the corresponding hypotheses.

## 2 Literature review and hypothesis development

### 2.1 AI acceptance in mathematics education

The Technology Acceptance Model (TAM) explains technology use primarily through perceived usefulness and perceived ease of use (Davis, 1989), and meta-analytic evidence supports its relevance to teachers' adoption of digital technologies (Scherer et al., 2019). Consistent with this framework, research applying TAM specifically to pre-service mathematics teachers' use of ChatGPT has shown that perceived ease of use and perceived usefulness significantly predicted attitudes toward the tool and its actual use, with pedagogical AI self-efficacy emerging as an important antecedent (Demir, 2026). TAM and related adoption frameworks distinguish technology-related beliefs and evaluations from use behavior (Davis, 1989; Venkatesh et al., 2003). Accordingly, actual use was treated here as a behaviorally distinct dimension rather than as an interchangeable indicator of psychological acceptance. GenAI, however, adds forms of uncertainty that are less prominent in many conventional educational technologies: outputs are probabilistic, their accuracy must be evaluated, and expectations for appropriate classroom use continue to change. These conditions do not invalidate TAM, but they suggest that teachers' acceptance may also be associated with how they cognitively adapt to, and respond to, technology-related strain. In this study, TAM is used as an organizing framework rather than being tested as a complete structural model or extended. In the revised analysis, perceived usefulness, perceived ease of use, attitude toward use, and pedagogical AI self-efficacy were combined into a study-specific ChatGPT acceptance composite, whereas actual use was excluded. This index is an aggregate indicator created to address the study's overall research question rather than a separately validated higher-order latent factor; consequently, the analysis does not estimate relations among the individual TAM components. The four retained dimensions were treated as conceptually distinguishable but complementary indicators of a broader psychological acceptance orientation. Their aggregation was therefore a substantive scoring decision aligned with the study's overall research question, not a claim that usefulness, ease of use, attitude, and pedagogical AI self-efficacy are interchangeable manifestations of one latent trait.

UTAUT and UTAUT2 broaden acceptance research by incorporating social influence, facilitating conditions, and individual differences (Venkatesh et al., 2003, 2012). Those constructs are relevant but fall outside the present model. Here, the narrower question is whether two psychological characteristics—cognitive flexibility and technostress—are associated with variation in teachers' overall acceptance of ChatGPT for mathematics instruction. This boundary is important: the study complements technology-acceptance theory with a psychological adaptation perspective, but it does not constitute a comparative test of TAM, UTAUT, or UTAUT2.

In the present study, AI acceptance is understood as teachers' favorable beliefs, evaluations, and capability perceptions concerning the integration of AI tools into instruction. It is therefore broader than a single judgment of usefulness, although utility and ease of use remain central components. Actual use was not included in the revised acceptance composite because it represents self-reported behavior rather than a belief, evaluation, or capability perception. Çakir and Güner (2026) similarly conceptualized teachers' AI acceptance as a multidimensional construct encompassing AI-use experience, perceived usefulness, perceived ease of use, behavioral intention, self-efficacy, and anxiety. Their findings showed high overall AI acceptance among 204 middle- and high-school teachers and a positive association between TPACK and AI acceptance. However, technostress was neither significantly correlated with nor predictive of this acceptance. This result reinforces the need to examine psychological predictors beyond competence and indicates that the technostress–acceptance relationship may be context-dependent.

Recent evidence shows that research on GenAI adoption in education remains dominated by conventional acceptance variables and higher-education student samples. In a systematic review of 43 empirical studies, Tan (2026) identified TAM as the most frequently used framework and noted that comparatively fewer studies focused on teachers or school settings. Teacher-focused studies similarly emphasize perceived usefulness, effort expectancy, social influence, facilitating conditions, competence, and self-efficacy. For example, AI-integration self-efficacy was the strongest predictor of K−12 teachers' attitudes toward GenAI in Darko et al. (2026), while AI-acceptance variables were positively associated with teachers' AI literacy, with hedonic motivation and willingness to use AI emerging as the strongest predictors (Deshen et al., 2026). These findings establish the relevance of acceptance constructs, but also leave the role of teachers' adaptive cognitive and stress-related characteristics less fully specified.

This limitation is particularly visible in mathematics education. Among 230 K−12 mathematics teachers, Wang et al. (2025) found that attitudes, subjective norms, and perceived behavioral control directly predicted GenAI use, whereas AI awareness operated through perceived usefulness, perceived ease of use, and attitudes. Trung et al. (2025) likewise reported that performance expectancy, effort expectancy, social influence, and trust were associated with Vietnamese mathematics teachers' intention to use ChatGPT. Demir (2026), working with pre-service mathematics teachers, extended TAM by including pedagogical AI self-efficacy. Qualitative evidence further shows that mathematics teachers simultaneously recognize ChatGPT's potential for planning, explanation, and personalized learning and express concerns about mathematical accuracy, reasoning, and pedagogical alignment (Busuttil and Calleja, 2025). Thus, mathematics-specific research has begun to identify determinants of acceptance, but it has mainly relied on technology-, competence-, and belief-centered explanations.

### 2.2 Cognitive flexibility and general technology-related technostress

Cognitive flexibility is the capacity to recognize alternatives, shift perspectives, and adapt responses when circumstances change (Dennis and Vander Wal, 2010). These capacities may be especially relevant when teachers encounter GenAI outputs that are fluent yet uncertain, inconsistent, or pedagogically unsuitable. A cognitively flexible teacher may be better able to reconsider an initial interpretation, compare alternative uses, and revise an instructional plan without treating the technology as uniformly beneficial or threatening. Consistent with this reasoning, cognitive flexibility has been associated with AI self-efficacy, AI acceptance, and lower AI anxiety (Kaya and Keskin, 2026). Cognitive flexibility should nevertheless be treated here as an individual-difference correlate rather than as a demonstrated cause of acceptance. The reverse direction is equally plausible on theoretical grounds: persistent technostress may itself constrain flexible responding, for instance, by narrowing attention to coping with immediate technical demands, so the association tested here should not be read as evidence that flexibility is the causal starting point.

Direct evidence concerning cognitive flexibility and technostress is still limited. Bali et al. (2024) examined both constructs within the same structural model and found that lower self-reported cognitive flexibility was associated with higher technostress; technostress also statistically linked cognitive flexibility with smartphone skills. From a stress-appraisal perspective, flexible individuals may generate alternative interpretations and coping responses when encountering technological uncertainty, thereby perceiving digital demands as more manageable (Lazarus and Folkman, 1984). Nevertheless, Bali et al.'s heterogeneous adult sample was not teacher-specific and focused on general ICT rather than GenAI. The cognitive flexibility–technostress association, therefore, remains insufficiently examined among practicing teachers and has not been established specifically among mathematics teachers using GenAI.

H1. Mathematics teachers' cognitive flexibility is negatively associated with their general technology-related technostress.

### 2.3 General technology-related technostress and AI acceptance

Technostress refers to strain associated with demands created by digital technologies (Tarafdar et al., 2007). For teachers, such demands can include continual learning, technical uncertainty, workload, role disruption, and perceived loss of control. GenAI can intensify these demands because teachers must monitor rapidly changing tools and evaluate outputs whose quality cannot be assumed. Higher technostress has been linked to lower perceived usefulness and lower continuance intentions regarding educational technologies (Wang and Yu, 2024). From a stress-appraisal perspective (Lazarus and Folkman, 1984), flexible appraisal may be associated with lower perceived strain, while greater strain may coincide with less favorable evaluations of ChatGPT.

Research on teacher technostress also cautions against assuming a uniform relationship with adoption. Joo et al. (2016) found that secondary teachers' technostress was negatively associated with their intention to use technology. Wang and Zhao (2023), however, showed that the associations depended on particular technostress creators: techno-complexity and techno-insecurity were negatively related to ICT attitudes and adoption intentions, whereas other dimensions displayed different patterns. Khlaif et al. (2023) found no direct association between technostress and K−12 teachers' continuance intention to use mobile technology, although indirect negative associations emerged through perceived usefulness and attitude. In the more recent AI context, Çakir and Güner (2026) found that TPACK predicted teachers' AI acceptance, but technostress did not. Meanwhile, Wu et al. (2026) documented that competence, role conflict, organizational support, and technology characteristics were associated with middle-school teachers' AI-related technostress. Collectively, these studies suggest that technostress is relevant to teachers' technological adaptation, but its association with acceptance may vary by technology, stress dimension, outcome, and context.

H2. Mathematics teachers' general technology-related technostress is negatively associated with their acceptance of ChatGPT for mathematics instruction.

### 2.4 Cognitive flexibility and AI acceptance

Emerging research suggests that cognitive flexibility is also relevant to how individuals evaluate and approach AI. Kaya and Keskin (2026) found that cognitive flexibility was positively associated with AI self-efficacy and acceptance and negatively associated with AI anxiety. Alshammari et al. (2026) extended UTAUT by incorporating cognitive flexibility into a model of students' AI adoption. Although cognitive flexibility did not directly predict behavioral intention, it showed a significant indirect association through performance expectancy, indicating that adaptive cognitive characteristics may complement expectancy-based explanations of AI adoption. In a teacher sample, Dere and Dogan (2025) reported a positive, although modest, association between perceptions of AI use in education and flexible thinking. Collectively, these studies support the relevance of cognitive flexibility to AI-related responses, although its association with acceptance may not always be direct. Nevertheless, evidence remains scarce regarding practicing mathematics teachers' acceptance of ChatGPT-specific instruction.

Cognitive flexibility may be especially relevant to GenAI because interaction with generative systems rarely follows a fully predictable sequence. Teachers may need to compare alternative prompts, shift between pedagogical and mathematical criteria, reconsider an initially plausible response, and revise an instructional plan when an output is inaccurate or unsuitable. Accordingly, flexibility may be associated with acceptance not simply because flexible individuals are more favorable toward technology, but because they may perceive a wider range of workable responses when an AI interaction fails or remains ambiguous. Evidence outside mathematics teaching supports this interpretation. Karaoglan Yilmaz and Yilmaz (2025) found that cognitive flexibility was positively associated with attitudes toward GenAI alongside self-regulated learning and metacognitive awareness. Similarly, Özsoy and Karakuş (2023) reported a positive relationship between cognitive flexibility and attitudes toward AI technologies among university students studying sports sciences. These findings involve different populations and attitude measures and, therefore, cannot be transferred directly to practicing mathematics teachers. Nevertheless, their convergence suggests that cognitive flexibility is a plausible person-level correlate of favorable AI evaluation and that it warrants tests specific to teachers and to ChatGPT.

H3. Mathematics teachers' cognitive flexibility is positively associated with their acceptance of ChatGPT for mathematics instruction.

### 2.5 The indirect role of general technology-related technostress and the present study

Bringing these perspectives together yields a bounded psychological adaptation model. From a person-environment fit perspective, technostress is theorized to arise when technological demands, such as complexity, unreliability, intrusiveness, or rapid change, exceed the resources available to manage them (Ayyagari et al., 2011). Cognitive flexibility may function as one such adaptive resource because generating alternative interpretations and switching responses may be associated with a lower likelihood that an uncertain AI interaction is appraised as unmanageable. Greater technostress may, in turn, coincide with lower acceptance of ChatGPT when strain co-occurs with lower perceived competence, perceived control, or willingness to engage with innovation. Adjacent evidence supports this link: Zhang (2023) found that technostress was negatively associated with academic self-efficacy and positively associated with resistance to innovation, with self-efficacy forming part of the statistical pathway. Although that study did not examine teachers or ChatGPT, it provides relevant evidence that technology-related strain can be connected to psychological processes associated with reluctance toward innovation. The resulting cognitive flexibility–technostress–acceptance pathway is therefore theoretically plausible; however, the present cross-sectional design can only establish whether the covariance pattern is consistent with that pathway. It cannot establish temporal ordering, rule out reverse or reciprocal relationships, or show that changing cognitive flexibility would alter technostress or acceptance.

Overall, the literature reveals three connected gaps. First, studies of mathematics teachers' GenAI adoption have largely concentrated on TAM, TPB, UTAUT, awareness, trust, and self-efficacy, rather than on integrating cognitive adaptability with technology-related strain. Second, the small emerging literature connects cognitive flexibility with AI attitudes, anxiety, and perceptions of AI use across student, adult, and teacher samples (Özsoy and Karakuş, 2023; Dere and Dogan, 2025; Karaoglan Yilmaz and Yilmaz, 2025; Kaya and Keskin, 2026), but direct evidence connecting cognitive flexibility to technostress remains limited and has not been established among practicing mathematics teachers in a GenAI context. Third, findings concerning technostress and teachers' acceptance, or continuance intention are mixed, including negative, indirect, dimension-specific, and non-significant associations. Existing studies also indicate that technostress may be linked with innovation resistance through diminished self-efficacy (Zhang, 2023), yet this mechanism has not been examined as part of a cognitive-flexibility model of teachers' ChatGPT acceptance. To our knowledge, no study has tested whether general technology-related technostress statistically accounts for part of the association between cognitive flexibility and ChatGPT acceptance among in-service mathematics teachers. The present study addresses this gap by estimating direct associations and a bootstrapped estimate of the indirect effect.

H4. Cognitive flexibility has a positive indirect statistical association with ChatGPT acceptance through general technology-related technostress.

## 3 Methods

This study used a cross-sectional, quantitative, correlational design. Cognitive flexibility was specified as the predictor, general technology-related technostress as the intervening variable, and the revised four-dimension ChatGPT acceptance composite as the outcome. Gender was included as a pre-specified covariate. Because the predictor, the intervening variable, and the outcome were measured simultaneously, the model estimates conditional associations and a statistical indirect effect rather than temporal or causal mediation.

### 3.1 Participants

The study sample consists of 127 middle school mathematics teachers working in approximately 30 public schools in the Marmara Region. Teachers were recruited with official permission from the relevant provincial and district directorates of national education and from school administrations. Invitations were distributed to mathematics teachers in the participating schools, and participation was voluntary. Only teachers currently employed to teach mathematics were included in the study; no additional exclusion criteria were applied. Of the 140 teachers who were invited and who received the questionnaire, 131 completed it (response rate = 93.6%). During data screening, multivariate outliers were identified using Mahalanobis distance (*p* < 0.001; Hair et al., 2019); four cases exceeded this threshold and were excluded from further analysis, resulting in a final analytic sample of 127 teachers. Data were collected from teachers through face-to-face surveys.

Of the participants, 67.7% (*n* = 86) were female teachers and 32.3% (*n* = 41) were male teachers. Examination of the age distribution showed that 14.2% of teachers were aged 25 and under, 44.1% were aged 26–35, 30.7% were aged 36–45, and 11% were aged 46 and over. Regarding professional seniority, 25.2% of teachers had 1–5 years of experience, 37% had 6–10 years, 24.4% had 11–15 years, and 13.4% had 16 or more years of experience.

In addition, participants were asked about the frequency of their use of artificial intelligence applications in their teaching practices. According to the responses, 34 teachers (26.77%) reported never using AI tools, another 34 (26.77%) reported rarely using them, 47 (37.01%) reported occasionally using them, and 12 (9.45%) reported frequently using them. These findings indicate that participants exhibited varying levels of familiarity with and prior experience using AI-based technologies. Participants were also asked whether they had received any formal training related to artificial intelligence tools. Of the 127 teachers, 37.80% (*n* = 48) reported having received such training, whereas 62.20% (*n* = 79) reported that they had not. Participants were further asked which AI applications they used in their professional practice, with multiple responses permitted. ChatGPT was reported by 72.44% of participants (*n* = 92), Gemini by 43.31% (*n* = 55), Claude by 13.39% (*n* = 17), Copilot by 3.94% (*n* = 5), and other applications by 1.57% (*n* = 2). Because participants could select more than one application, the percentages do not sum to 100%. This distribution indicates that ChatGPT was the most commonly reported application, although many teachers also used other generative AI tools; this pattern is relevant to interpreting the ChatGPT-specific acceptance measure employed in this study.

### 3.2 Procedure and ethical considerations

Data were collected during November and December 2025. Participation was voluntary, and participants were informed of the study's purpose prior to data collection. The questionnaires were administered face-to-face in school settings. Participants completed the questionnaires anonymously, and no personally identifiable information was collected. Confidentiality and anonymity were assured throughout the study. Ethical approval for the study was obtained in 2025 from the relevant institutional ethics committee (Approval No: E-95104632-204.01.07-706043).

### 3.3 Data collection tools

#### 3.3.1 Techno-stress scale

The Techno-stress Scale developed by Çoklar et al. (2017) was used to determine participants' technology-related stress levels. The scale was developed to measure teachers' technology-related stress in educational settings. The scale consists of five dimensions—learning-teaching process, career-related, technical, personal, and social—and includes a total of 28 items. Participants responded to the statements using a 5-point Likert-type scale (1 = Strongly Disagree, 5 = Strongly Agree). In the original study, Cronbach's alpha coefficients for the five dimensions ranged from 0.712 to 0.788, while the overall scale demonstrated high internal consistency (α = 0.917). The Spearman–Brown coefficient was 0.845. An example item is: “I think technology use makes teaching profession more difficult.” The scale was developed prior to the emergence of generative AI tools and addresses general technology-related stress among teachers rather than stress specific to AI or GenAI use; its items do not explicitly reference ChatGPT or similar generative systems. The scale was selected because technostress was conceptualized in the present study as teachers' broader strain in adapting to changing digital technologies rather than as a ChatGPT-specific reaction. Accordingly, the scale was used as a measure of general technostress that may be relevant to ChatGPT acceptance, not as a measure of ChatGPT-specific technostress.

#### 3.3.2 Cognitive flexibility inventory

The Cognitive Flexibility Inventory, developed by Dennis and Vander Wal (2010) and adapted into Turkish by Sapmaz and Dogan (2013), was used to measure participants' abilities to consider different perspectives, develop alternative solutions, and perceive difficult situations as controllable. The Turkish version consists of 20 items and uses a 5-point Likert-type response format (1 = Strongly Disagree, 5 = Strongly Agree). In the Turkish adaptation study, Cronbach's alpha was .90 for the overall scale, .90 for the Alternatives dimension, and .84 for the Control dimension. An example item, translated from the Turkish form, is “I believe that every problem has more than one solution.”

#### 3.3.3 Technology acceptance scale for the use of artificial intelligence tools in mathematics lessons

The Technology Acceptance Scale for the Use of Artificial Intelligence (ChatGPT) in Mathematics Lessons, developed by Demir (2026), was used to assess participants' acceptance of ChatGPT for mathematics instruction. The original scale consists of 18 items across five dimensions: perceived usefulness, perceived ease of use, attitude toward use, actual use, and pedagogical AI self-efficacy. Items are rated on a 5-point Likert-type scale (1 = Strongly Disagree, 5 = Strongly Agree). In the original study, Cronbach's alpha coefficients ranged from .679 to .853 across the five dimensions, with additional evidence supporting the instrument's construct validity. An example item is: “Using ChatGPT in mathematics lessons makes my teaching tasks easier.” For the revised analysis, the actual-use dimension was excluded from the acceptance composite because it represents self-reported behavior, whereas perceived usefulness, perceived ease of use, attitude toward use, and pedagogical AI self-efficacy represent beliefs, evaluations, or capability perceptions. The ChatGPT acceptance score was therefore calculated from these four dimensions. Specifically, responses to the retained items were averaged without differential weighting to form the study-specific composite; higher values indicated a more favorable overall psychological acceptance orientation. This transparent unit-weighting procedure was used to provide a parsimonious summary for the focal indirect-effect analysis rather than to estimate a latent higher-order score. This approach does not imply that the four dimensions constitute a single higher-order latent factor and does not permit dimension-specific inferences. Accordingly, the outcome labeled AI Acceptance in Figure 1 refers specifically to this study-specific composite of ChatGPT acceptance for mathematics instruction, rather than acceptance of AI technologies in general. Actual use was not modeled as a separate behavioral outcome, and dimension-specific analyses were not conducted; both issues are addressed as limitations below. Although the scale was originally developed and validated with pre-service mathematics teachers using PLS-SEM (Demir, 2026), the original study recommended extending its application to practicing teachers in authentic classroom settings. Given this population difference and the revised outcome specification, the four-factor measurement structure was evaluated with confirmatory factor analysis in the present in-service teacher sample (Table 1).

**Figure 1:**
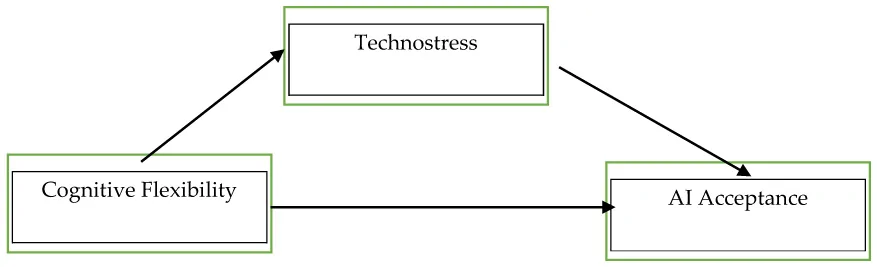
The research model.

**Table 1:** CFA fit indices, factor loadings, CR, and AVE values.

Scale	χ^2^/df	CFI	GFI	RMSEA	SRMR	Loadings	CR	AVE
Cognitive flexibility inventory	1.956	0.912	0.923	0.069	0.057	0.584–0.788	0.845	0.464
Technostress scale	2.123	0.902	0.907	0.077	0.065	0.544–0.715	0.806	0.506
ChatGPT acceptance scale for mathematics lessons	2.050	0.925	0.916	0.070	0.056	0.705–0.781	0.883	0.654

### 3.4 Data analysis

Data were analyzed using IBM SPSS Statistics 29, IBM SPSS AMOS, and PROCESS v4.1 (Hayes, 2022). The dataset was screened before hypothesis testing. No values were missing. Four multivariate outliers were excluded, yielding the final analytic sample of 127. Skewness and kurtosis values were between −2 and +2; residual plots and scatterplots did not reveal substantial departures from linearity or homoscedasticity; and multicollinearity diagnostics were acceptable (VIF < 3; tolerance > 0.20). A sensitivity analysis indicated that, at α = 0.05 and power = 0.80, the two- and three-predictor regression models could detect effects of approximately f^2^ = 0.078 and 0.089, respectively (minimum detectable R^2^ ≈ 0.07–0.08). This sensitivity result describes the effects detectable with the obtained sample and should not be interpreted as proof of adequate precision for every parameter.

Separate confirmatory factor analyses were conducted for the three instruments using IBM SPSS AMOS with maximum likelihood estimation. Consistent with their analyzed structures, the Cognitive Flexibility Inventory was specified as a two-factor model, the Technostress Scale as a five-factor model, and the revised ChatGPT acceptance measure as a four-factor model with correlated first-order factors after exclusion of the actual-use dimension. The five-point Likert items were treated as continuous observed variables. This pragmatic analytic choice was based on the five-category response format and the limited, sample-specific purpose of the CFAs rather than on an assumption that the items were intrinsically continuous. The model-fit indices (χ^2^/df, CFI, GFI, RMSEA, and SRMR) and standardized loading ranges are reported in Table 1. Because the models retained correlated first-order dimensions, a single overall CR or AVE value is not interpreted as evidence of a higher-order factor. The internal consistency of the analyzed composite scores is reported separately in Table 2 using Cronbach's alpha. Across the three models, all reported standardized loadings exceeded .50 (overall range = 0.544–0.788), supporting retention of the analyzed indicators. Given *N* = 127 and the instruments' length and multidimensionality, these CFAs should be regarded as sample-specific supporting evidence rather than definitive revalidation. Traditional guidelines recommend a minimum of 100 participants and approximately five participants per variable for factor analysis (Gorsuch, 1983). However, more recent simulation-based evidence indicates that sample-size requirements for CFA depend on model complexity, factor loadings, and the number of indicators rather than on a single universal threshold (Wolf et al., 2013). Accordingly, the present sample (*N* = 127) exceeded the traditional minimum sample size, although the CFA findings should be interpreted as sample-specific supporting evidence. Composite scores were used because the substantive model was concerned with overall cognitive flexibility, technostress, and the study-specific four-dimension ChatGPT acceptance composite. Combining the four acceptance dimensions precludes conclusions about distinct TAM components and does not imply a single higher-order acceptance factor. The CFA and the regression analysis thus served different purposes: the four-factor CFA evaluated whether the retained component dimensions remained empirically distinguishable, whereas the equally weighted composite summarized their overall level for the pre-specified substantive model. Evidence for correlated first-order factors is therefore not presented as psychometric proof of unidimensionality.

**Table 2:** Descriptive statistics, reliability coefficients, and correlations among variables.

Variable	Cronbach's α	*M*	SD	Skewness	Kurtosis	1	2	3
1. Cognitive Flexibility	0.791	3.03	0.82	−0.52	−0.47	1		
2. Technostress	0.873	3.63	0.51	−0.43	0.31	−0.34^**^	1	
3. AI (ChatGPT) Acceptance	0.858	2.74	0.77	0.01	−0.15	0.50^**^	−0.62^**^	1

^**^p < 0.01 (two-tailed). CF, Cognitive Flexibility; TS, General Technology-Related Technostress; CA, ChatGPT Acceptance.

Internal consistency was evaluated using Cronbach's alpha. Gender was dummy-coded (0 = female, 1 = male) and included as a pre-specified covariate in both regression equations; it was not a focal theoretical variable. Further gender-based subgroup analyses were not conducted because they fell outside the scope of the study's hypotheses. The hypothesized indirect association was estimated with PROCESS Model 4 using 5,000 bootstrap samples and a 95% confidence interval. The primary model used the revised four-dimension ChatGPT acceptance composite that excluded actual use. The original five-dimension specification was retained as a robustness comparison. Unstandardized coefficients (B), rather than standardized β coefficients, are reported. An indirect effect was considered statistically distinguishable from zero when its bootstrap confidence interval excluded zero. Because all constructs were measured concurrently, the analysis is described as an indirect-effect analysis; the term mediation refers only to the specified statistical decomposition. Same-source, self-report measurement also creates a risk of common-method variance. Harman's single-factor diagnostic showed that the first unrotated factor accounted for approximately 32% of the total variance, which is below the conventional 50% threshold used to flag a dominant single factor. This diagnostic is nevertheless a weak safeguard: it can fail to detect common-method variance even when present; it does not model a method factor directly; and it cannot rule out method-related inflation of the observed associations, particularly given that all three constructs were assessed via the same self-report instrument administered in a single sitting. The possibility of common-method bias, therefore, remains a limitation that a stronger design (e.g., a marker-variable technique or a modeled common-method latent factor) would need to address.

## 4 Results

Table 2 reports descriptive statistics, internal-consistency estimates, and zero-order correlations.

Cognitive flexibility averaged 3.03 (SD = 0.82), general technology-related technostress averaged 3.63 (SD = 0.51), and ChatGPT acceptance averaged 2.74 (SD = 0.77). Cognitive flexibility was negatively correlated with general technology-related technostress (r = −0.34, *p* < 0.01) and positively correlated with ChatGPT acceptance (r = 0.50, *p* < 0.01). General technology-related technostress was negatively correlated with ChatGPT acceptance (r = −0.62, *p* < 0.01). These zero-order associations were consistent with H1–H3 and warranted estimation of the pre-specified indirect-effect model; they do not, by themselves, indicate directionality or causation.

Table 2 indicates broadly acceptable, sample-specific model fit for the three instruments, with CFI values ranging from 0.902 to 0.925, GFI values from 0.907 to 0.923, RMSEA values from 0.069 to 0.077, and SRMR values from 0.056 to 0.065. All standardized factor loadings exceeded 0.50 (range: 0.544–0.788), and CR values ranged from 0.806 to 0.883. For the Technostress Scale and the revised four-factor ChatGPT Acceptance Scale, AVE values exceeded 0.50, providing supportive evidence for convergent validity in this sample. For the Cognitive Flexibility Inventory, however, AVE was 0.464, below the conventional 0.50 threshold (Fornell and Larcker, 1981). Although its CR value (0.845) and loading range (0.584–0.788) provide acceptable supporting evidence for reliability and indicator performance, the evidence for convergent validity should be regarded as mixed rather than conclusive. Given the modest sample size (N = 127) relative to the length and multidimensionality of the instruments, the CFA findings are interpreted as provisional, sample-specific support and not as definitive validation of the measurement models. For the Cognitive Flexibility Inventory specifically, future replications with larger samples could also consider item-parceling approaches, which tend to stabilize factor loadings and AVE estimates for lengthy, multidimensional instruments.

### 4.1 Analyses related to hypotheses

Table 3 presents the two regression equations and the bootstrapped indirect effect for the revised ChatGPT acceptance composite, which excludes actual use. Coefficients are unstandardized. The first equation estimated general technology-related technostress from cognitive flexibility and gender; the second estimated ChatGPT acceptance from cognitive flexibility, general technology-related technostress, and gender.

**Table 3:** Regression coefficients and the bootstrapped indirect effect for ChatGPT acceptance.

Outcome	Predictor	*R^2^*	*F*	*B*	SE	Lower	Upper	*t*	*p*
TS	CF	0.189	14.4	−0.243	0.052	−0.346	−0.139	−4.641	< 0.001
	Gender			0.330	0.102	0.128	0.531	3.242	0.002
CA	CF (direct)	0.526	45.5	0.355	0.063	0.230	0.480	5.635	< 0.001
	TS			−0.620	0.101	−0.820	−0.420	−6.139	< 0.001
	Gender			−0.470	0.117	−0.702	−0.238	−4.017	< 0.001
Indirect association	CF → TS → CA			0.151	0.043	0.074	0.241		
Total association	CF → CA			0.506	0.091	0.326	0.686	5.560	< 0.001

CF, Cognitive Flexibility; TS, General Technology-Related Technostress; CA, ChatGPT Acceptance.

Cognitive flexibility was negatively associated with general technology-related technostress after adjustment for gender (B = −0.243, SE = 0.052, 95% CI [−0.346, −0.139], *p* < 0.001), supporting H1. General technology-related technostress was negatively associated with ChatGPT acceptance after adjustment for cognitive flexibility and gender (B = −0.620, SE = 0.101, 95% CI [−0.820, −0.420], *p* < 0.001), supporting H2. Cognitive flexibility retained a positive conditional association with ChatGPT acceptance (B = 0.355, SE = 0.063, 95% CI [0.230, 0.480], *p* < 0.001), supporting H3. These coefficients quantify conditional mean differences in this sample and should not be interpreted as effects of changing either construct. The total association between cognitive flexibility and ChatGPT acceptance was positive and statistically significant (B = 0.506, SE = 0.091, 95% CI [0.326, 0.686], t = 5.560, *p* < 0.001). The bootstrapped indirect effect of cognitive flexibility on ChatGPT acceptance through general technology-related technostress was positive (B = 0.151, SE = 0.043, 95% CI [0.074, 0.241**]**), supporting H4 as an indirect statistical association. The direct association remained positive and statistically significant; a pattern conventionally described as partial statistical mediation. The outcome model explained 52.6% of the variance in ChatGPT acceptance.

As a robustness comparison, the original five-dimension specification that included actual use yielded an indirect association of B = 0.142, SE = 0.042, 95% CI [0.069, 0.230]. The direction and statistical significance of the findings were therefore unchanged, indicating that the principal result was not dependent on including actual use in the composite outcome. Gender was also associated with general technology-related technostress and ChatGPT acceptance. Based on the stated coding, men reported higher general technology-related technostress (B = 0.330, *p* = 0.002) and lower ChatGPT acceptance (B = −0.470, *p* < 0.001). Because gender was not a focal hypothesis and potentially relevant covariates were unavailable, these conditional differences are descriptive and should be interpreted cautiously.

Two further caveats bear directly on these estimates. First, because the intraclass correlation for teachers nested within schools could not be computed and clustering was not modeled, the standard errors, confidence intervals, and *p*-values reported above assume independent observations and may be somewhat anti-conservative. Second, gender was the only covariate available; the possibility that an unmeasured characteristic (e.g., prior AI training, digital competence, or school-level support) confounds one or more of the reported associations cannot be ruled out. Both estimates should therefore be read as suggestive rather than as precise, confound-free effect sizes.

## 5 Discussion

This study examined whether general technology-related technostress statistically accounted for part of the association between cognitive flexibility and mathematics teachers' acceptance of ChatGPT. The observed pattern was consistent with the proposed psychological adaptation model: greater cognitive flexibility was associated with lower general technology-related technostress and higher ChatGPT acceptance, while general technology-related technostress was associated with lower ChatGPT acceptance. A positive bootstrapped indirect statistical association remained after adjustment for gender. The revised model, in which actual use was excluded from the ChatGPT acceptance composite, explained 52.6% of the variance in ChatGPT acceptance. A robustness comparison using the original five-dimension composite that included actual use produced findings with the same direction and statistical significance. These findings add a psychological adaptation perspective to technology-acceptance research by showing that teachers' evaluations of GenAI coexist with individual differences in cognitive flexibility and general technology-related strain. They do not constitute a structural test or an extension of TAM because its component constructs were not separately modeled. Temporal or causal mediation cannot be established because all variables were assessed at one time point.

### 5.1 The relationship between cognitive flexibility and general technology-related technostress

The negative association between cognitive flexibility and technostress provides teacher-specific evidence, in a context involving GenAI adoption, of a relationship that has previously been tested mainly in broader digital contexts. Because the technostress measure assessed general technology-related strain rather than ChatGPT- or GenAI-specific strain (see Section 3.3.1), this evidence should be read as extending the flexibility–technostress link to teachers coping with a changing digital environment that includes GenAI, not as demonstrating a link specific to ChatGPT-related strain. Bali et al. (2024) found that lower self-reported cognitive flexibility was associated with greater technostress, and that technostress statistically connected cognitive flexibility with smartphone skills. The present result extends that pattern to practicing mathematics teachers working in a changing digital environment that includes ChatGPT. It is also consistent with adjacent AI evidence: cognitive flexibility has been positively associated with AI acceptance and self-efficacy and negatively associated with AI anxiety (Kaya and Keskin, 2026), while teachers' perceptions of AI use in education have been positively associated with flexible thinking (Dere and Dogan, 2025). Stress-appraisal theory offers a plausible interpretation: teachers able to generate alternative appraisals and responses may experience uncertain outputs, changing interfaces, and instructional disruption as more manageable (Lazarus and Folkman, 1984). Nevertheless, cross-sectional results cannot establish that flexibility reduces technostress; lower strain or prior successful AI experience could instead account for more flexible self-perceptions.

### 5.2 The relationship between general technology-related technostress and AI acceptance

The negative conditional association between general technology-related technostress and ChatGPT acceptance is consistent with Joo et al. (2016), who reported that secondary teachers' technostress was associated with lower intention to use technology. It is also compatible with evidence that techno-insecurity was negatively associated with teachers' attitudes toward ICT, whereas techno-complexity was negatively associated with their ICT adoption intentions (Wang and Zhao, 2023). In mathematics teaching, the need to verify symbolic accuracy, logical reasoning, solution validity, and pedagogical appropriateness may make ChatGPT-related demands especially salient; qualitative work documents teachers' simultaneous recognition of the tool's potential and concern about its mathematical limitations (Busuttil and Calleja, 2025). However, the literature is not uniform. Khlaif et al. (2023) found no direct technostress–continuance relationship among K−12 teachers, although indirect associations occurred through usefulness and attitude, and Çakir and Güner (2026) found that technostress neither correlated significantly with nor predicted teachers' AI acceptance. Our finding should therefore be interpreted as context-specific evidence rather than a universal effect. Differences in the focal technology, technostress operationalization, teacher group, outcome definition, and model covariates may explain the divergent results.

An important qualification concerns instrument specificity. The Techno-stress Scale (Çoklar et al., 2017) was developed before generative AI tools existed and captures general technology-related strain—covering learning-teaching, career, technical, personal, and social dimensions—rather than reactions specific to ChatGPT or other GenAI tools. Consequently, the negative technostress–acceptance association reported here indicates that teachers' broader digital strain co-occurs with lower ChatGPT acceptance; it does not establish that strain generated specifically by ChatGPT use is what drives this association. Two competing interpretations remain open. On one hand, general technostress may act as a stable individual-difference proxy that generalizes to how teachers appraise any new technology, including ChatGPT, in which case the present association may reasonably approximate a ChatGPT-relevant relationship. On the other hand, a GenAI-specific technostress measure might reveal a stronger, weaker, or differently patterned association, since ChatGPT introduces distinct demands—such as verifying probabilistic and occasionally inaccurate outputs—that a general ICT scale was not designed to capture. The present data cannot adjudicate between these possibilities, and this measurement mismatch should be treated as an open question for GenAI-specific instrumentation rather than as evidence either supporting or undermining the observed association.

The gender pattern observed in the present study, with men reporting higher general technology-related technostress and lower ChatGPT acceptance, is directionally consistent with Çakir and Güner (2026), who reported significantly higher AI acceptance among women and higher, although non-significant, mean general technology-related technostress among men. It is also consistent with Wu et al. (2026), who found significantly higher technostress among male than female middle-school teachers. However, gender-related findings concerning teachers' attitudes toward, and acceptance of AI and other technologies have not been consistent across studies and may vary according to the technology, educational context, and outcome examined. Because the sample was predominantly female (67.7%) and gender was included only as a covariate rather than a focal variable, the observed associations—particularly the lower ChatGPT acceptance among men—should be interpreted as descriptive of this sample rather than as generalizable gender differences. Dedicated research with more balanced samples is needed to examine these patterns.

AI-use frequency provides important context about the sample rather than serving as an additional hypothesis test. In the present sample, 73.23% of participants reported at least some use of AI tools. This finding broadly aligns with evidence that GenAI is becoming increasingly visible in teachers' professional practice across different educational contexts (Pettersson et al., 2024; Gallup, 2025; Picton and Clark, 2025). Direct comparisons are not warranted because these studies differ by country, teacher groups, and measurement approaches. Moreover, the percentage observed in the present study should not be interpreted as a ChatGPT-specific adoption rate, because the frequency item referred broadly to AI tools rather than to ChatGPT specifically.

The mean revised ChatGPT acceptance composite was 2.74 on the five-point response scale. Because actual use was excluded from this composite, the acceptance score should be interpreted as reflecting perceived usefulness, perceived ease of use, attitude toward use, and pedagogical AI self-efficacy rather than behavioral adoption. The broad AI-use frequency item and the ChatGPT-specific acceptance composite were not analyzed at the individual level; therefore, their descriptive results should not be interpreted as evidence that exposure corresponded to acceptance. Teachers may try or use AI intermittently while retaining reservations about its usefulness, reliability, ease of use, pedagogical fit, or institutional conditions. This interpretation is consistent with Velli and Zafiropoulos (2024), who showed that perceived usefulness, social influence, and personal innovativeness were directly associated with teachers' behavioral intention to use educational AI tools, whereas trust and constructivist pedagogical beliefs were indirectly associated with intention through perceived usefulness. It also accords with mathematics-teacher research showing that favorable perceptions may not translate directly into use because subjective norms and perceived behavioral control are also associated with teachers' GenAI usage behavior (Wang et al., 2025). In the present study, the associations involving cognitive flexibility and general technology-related technostress suggest that variation in acceptance is connected not only to the technology's features but also to how its demands are cognitively appraised and psychologically experienced. Because the measures were cross-sectional and actual use was not modeled as a separate outcome, these findings do not establish that psychological factors cause behavioral adoption.

### 5.3 The indirect role of general technology-related technostress

The bootstrapped indirect effect combines two largely separate strands of research: one linking cognitive flexibility to more favorable AI-related responses, and the other linking technology-related strain to teachers' adoption. The finding suggests that general technology-related technostress statistically accounts for part of the positive association between cognitive flexibility and ChatGPT acceptance. This extends Bali et al. (2024) general-ICT evidence by placing the flexibility–technostress pathway within a teacher- and subject-specific acceptance model. It also complements mathematics-teacher studies centered on awareness, attitudes, subjective norms, behavioral control, expectancy, trust, and self-efficacy (Demir, 2026; Trung et al., 2025; Wang et al., 2025). The remaining direct association indicates that general technology-related technostress is not the only statistical pathway; flexible teachers may also more readily evaluate alternative instructional uses, recover from unsuccessful prompts, or revise lesson plans. However, because all variables were measured concurrently this pattern is compatible with—but does not verify—the proposed sequence. Longitudinal and experimental research are needed to distinguish it from reverse, reciprocal, and omitted-variable explanations.

### 5.4 Educational and practical implications

The findings support the following cautious practical implication: professional learning initiatives for GenAI should attend to psychological demands as well as to technical skills. Training could provide opportunities to evaluate imperfect GenAI-generated mathematical content, compare alternative prompts or solutions, and discuss workload and uncertainty. Such initiatives could also draw on broader evidence regarding protective factors against technostress among teachers (Zivi et al., 2025). Such activities are theoretically relevant to flexible appraisal and technostress, but this study did not test an intervention and cannot show that flexibility workshops, workload adjustments, or institutional support would increase acceptance. Their effectiveness should be evaluated experimentally or longitudinally before prescriptive recommendations are made.

### 5.5 Limitations and future research

Several limitations define the scope of the present findings. First, the cross-sectional design cannot establish temporal ordering or causal mediation; thus, reverse, reciprocal, and omitted-variable explanations remain possible. Second, all constructs were measured using self-reported data collected from the same respondents. Common-method variance and response tendencies may therefore have inflated the observed associations, and Harman's single-factor diagnostic cannot definitively rule out this possibility. Third, the voluntary sample was modest in size and was drawn from approximately 30 public schools in a single region of Türkiye. Moreover, because questionnaires were administered anonymously and no school-identifying information was collected, the intraclass correlation for teachers nested within schools could not be computed, and the nested teacher-within-school structure was not modeled. With an average of roughly four teachers per school, some clustering of responses is plausible, which may have led to standard errors in the regression estimates being somewhat underestimated, meaning that some of the reported confidence intervals and *p*-values may appear more precise or more statistically significant than the true, clustering-adjusted sampling variability would support. As an illustrative check rather than a formal correction, assuming a conservative intraclass correlation of .05 for an average cluster size of four teachers per school implies a design effect of approximately 1.15, corresponding to roughly a 7% inflation in standard errors; this suggests that any underestimation is likely to be modest rather than severe, though it cannot be verified without school-identifying data; this limits both generalizability and the precision of measurement and of the regression estimates. Fourth, the acceptance instrument referred specifically to ChatGPT, whereas the use-frequency item referred more broadly to AI applications. Fifth, and among the more consequential limitations, the revised composite acceptance score aggregates four conceptually distinct dimensions—perceived usefulness, perceived ease of use, attitude, and pedagogical AI self-efficacy—into a single index. Cognitive flexibility and technostress could plausibly relate differently, in direction or magnitude, to these dimensions; the present design cannot detect, rule out, or quantify such divergence, and no dimension-specific supplementary analysis was conducted. Consequently, the reported associations should be understood as pertaining only to teachers' overall acceptance orientation toward ChatGPT, not to any individual acceptance dimension, and the study should not be interpreted as providing a structural test of TAM component relationships. The use of the aggregate outcome consequently prioritizes parsimony and alignment with the overall research question at the cost of masking potentially different dimension-specific associations. Actual use was excluded from the revised composite because it represents self-reported behavior, and the robustness comparison showed that its exclusion did not alter the direction or significance of the principal findings. Nevertheless, actual use was not examined as a separate behavioral outcome; therefore, the present study cannot determine whether cognitive flexibility and technostress exhibit the same pattern of associations with actual ChatGPT use. Sixth, and this raises a specific omitted-variable concern, gender was the only covariate included in the model. Potentially relevant characteristics—age, professional seniority, prior AI training, tool-specific experience, digital competence, school resources, and organizational support—were not examined and could confound one or more of the reported associations; the magnitude, and possibly even the direction, of some coefficients could differ once such characteristics are accounted for. Seventh, the usage data indicated that a substantial proportion of participants primarily used AI applications other than ChatGPT, including Gemini. This mismatch should be considered when interpreting findings based on the ChatGPT-specific acceptance measure. Eighth, the technostress instrument assessed general technology-related strain rather than technostress arising specifically from GenAI tools. It therefore remains unclear to what extent the measured technostress reflected teachers' experiences with ChatGPT or other generative AI applications. Finally, the five-point Likert indicators were treated as continuous and estimated with conventional maximum likelihood. Because an ordinal CFA based on polychoric correlations or a robust categorical estimator was not available as a sensitivity analysis, the measurement results may partly depend on this analytic choice and should be interpreted cautiously.

Future research should employ longitudinal or cross-lagged designs to examine temporal ordering and competing directional explanations. Studies involving larger and more diverse samples should also account for the multilevel structure of teachers nested within schools. Tool-specific usage and acceptance measures, GenAI-specific technostress instruments, a broader set of individual and institutional covariates, separate modeling of the acceptance dimensions, and analysis of actual use as a distinct behavioral outcome would provide more precise evidence. Preregistered interventions could further examine whether changes in cognitive flexibility or technology-related support are accompanied by subsequent changes in technostress and AI acceptance.

## 6 Conclusions

Among practicing middle-school mathematics teachers, cognitive flexibility, general technology-related technostress, and the revised ChatGPT acceptance composite exhibited a coherent pattern of conditional associations. Teachers who reported greater cognitive flexibility also tended to have lower general technology-related technostress and greater acceptance; general technology-related technostress statistically accounted for part of this association. Excluding actual use from the acceptance composite did not alter the direction or statistical significance of the principal findings. The findings position psychological adaptation as a relevant complement to technology-centered explanations of GenAI acceptance. Their value lies in identifying a testable process for future research rather than in demonstrating that cognitive flexibility reduces technostress or causes acceptance of ChatGPT. Stronger longitudinal, multilevel, and intervention designs are required before causal or prescriptive conclusions are warranted.

## Data Availability

The raw data supporting the conclusions of this article will be made available by the authors, without undue reservation.
